# Visible-Light-Stimulated Synaptic Phototransistors Based on CdSe Quantum Dot/In–Ga–Zn–O Hybrid Channels

**DOI:** 10.1186/s11671-022-03739-8

**Published:** 2022-10-27

**Authors:** En-bo Fu, Yu Liu, Xiang-Rui Hou, Ye Feng, Chun-lei Yang, Yan Shao

**Affiliations:** 1 Shenzhen Institute of Advanced Technology, Chinese Academy of Sciences, Shenzhen, 518055 China; 2 Nano Science and Technology Institute, University of Science and Technology of China, Suzhou, 215123 China

**Keywords:** Synaptic phototransistors, CdSe quantum dot, In–Ga–Zn–O, Hybrid semiconductor film, Synaptic behaviors

## Abstract

Light-stimulated synaptic devices are promising candidates for the development of artificial intelligence systems because of their unique properties, which include broad bandwidths, low power consumption, and superior parallelism. The key to develop such devices is the realization of photoelectric synaptic behavior in them. In this work, visible-light-stimulated synaptic transistors based on CdSe quantum dot (CdSe QD)/amorphous In–Ga–Zn–O hybrid channels are proposed. This design can not only improve the charge separation efficiency of the photogenerated carriers, but also can induce delayed decay of the photocurrent. The improved charge separation efficiency enhances the photoelectric properties significantly, while the delayed decay of the photocurrent led to the realization of photoelectric synaptic behaviors. This simple and efficient method of fabricating light-stimulated phototransistors may inspire new research progress into the development of artificial intelligence systems.

## Introduction

The human brain is an ultra-high-performance network that consists of ~ 10^11^ neurons connected via ~ 10^15^ synapses, and it shows large advantages over the Von Neumann system when handling imprecisely defined problems [1–3]. As the basic units of the human brain, synapses offer distributed computation and combine computation and memory functions together. Therefore, the key step toward development of artificial intelligence systems is to fabricate high-performance synaptic devices [4]. Traditional synaptic devices can only process electrical signals, thus meaning that additional sensors are required when the input is optical signals [2, 5]. Photoelectric synaptic devices can not only combine light perception, signal processing, and data memory functions together, but also provide unique advantages, including broader bandwidths, high robustness, and superior parallelism [6, 7]. Therefore, these devices are regarded as the most suitable synaptic devices for simulation of the retinal neurons in human eyes.

To date, various types of materials have been used to fabricate photoelectric synaptic devices, including grapheme, MoS_2_, pentacene, perovskite materials, and amorphous In–Ga–Zn–O (a-IGZO) [8–13]. Among these materials, a-IGZO shows the highest potential for mass production because of its high electron mobility, large size uniformity, high stability, and low cost [14]. However, to simulate the human visual system, the synaptic devices must be sensitive to visible light, whereas a-IGZO cannot be used directly in this range because of its large bandgap (~ 3.1 eV). To overcome this disadvantage, additional light absorbers, such as quantum dots, have been used and led to enhanced photoelectric properties [15, 16]. In this work, phototransistors with high visible-light sensitivity were fabricated successfully using CdSe quantum dot (QD)/a-IGZO hybrid channels, in which the CdSe QDs served as visible-light absorbers. These phototransistors exhibited excellent photoelectric properties including a photocurrent-to-dark current (*I*_photo_/*I*_dark_) switching ratio of 1.8 × 10^5^, a high photoresponsivity (*R*) of 2.4 × 10^3^ A/W, and a high detectivity (D*) of 3.5 × 10^15^ Jones. Furthermore, typical photoelectric synaptic behaviors, including excitatory postsynaptic current (EPSC), paired-pulse facilitation (PPF), short-term plasticity (STP), and long-term plasticity (LTP), were also realized in these devices. This work provides a simple but effective method to fabricate photoelectric synaptic transistors, and may inspire new research progress toward the development of artificial intelligence systems.

## Experimental Details

A highly doped p-type silicon wafer (< 0.0015 Ω cm) was cleaned using wafer standard clean processes (SC-1) and was used as both the substrate and the gate electrode. A 40-nm-thick Al_2_O_3_ thin film was deposited using trimethylaluminum (TMA) as a precursor and H_2_O as an oxidant in an atomic layer deposition (ALD) system at 200 °C. An ALD process was consisted of a TMA pulse, a N_2_ purge, a H_2_O pulse, and a N_2_ purge, while their pulse times were set at 20 ms, 20 s, 20 ms, and 20 s, respectively. Then, an IGZO thin film was deposited by magnetron sputtering with the chamber pressure set at 0.3 Pa. Cr/Au (30 nm/70 nm) electrodes were deposited using electron beam evaporation. The channel area of 100 × 100 μm^2^ was defined via a lift-off process. The CdSe QDs were synthesized by the hot injection method using CdO and diphenylphosphine selenide (DPPSe) as precursors at 155 °C. The QDs obtained were purified with ethanol, dried, and then dissolved in octadecene (ODE). The synaptic transistors were then fabricated by spin coating the CdSe QD solution onto the a-IGZO channel area at 1500 rpm for 20 s. The photoelectric properties of the resulting devices were then measured using a semiconductor characterization analyzer (Keithley 4200 SC) in air at room temperature. Fiber-coupled laser modules were used to provide light inputs at wavelengths of 400 nm, 532 nm, and 635 nm.

## Results and Discussion

**Fig. 1:**
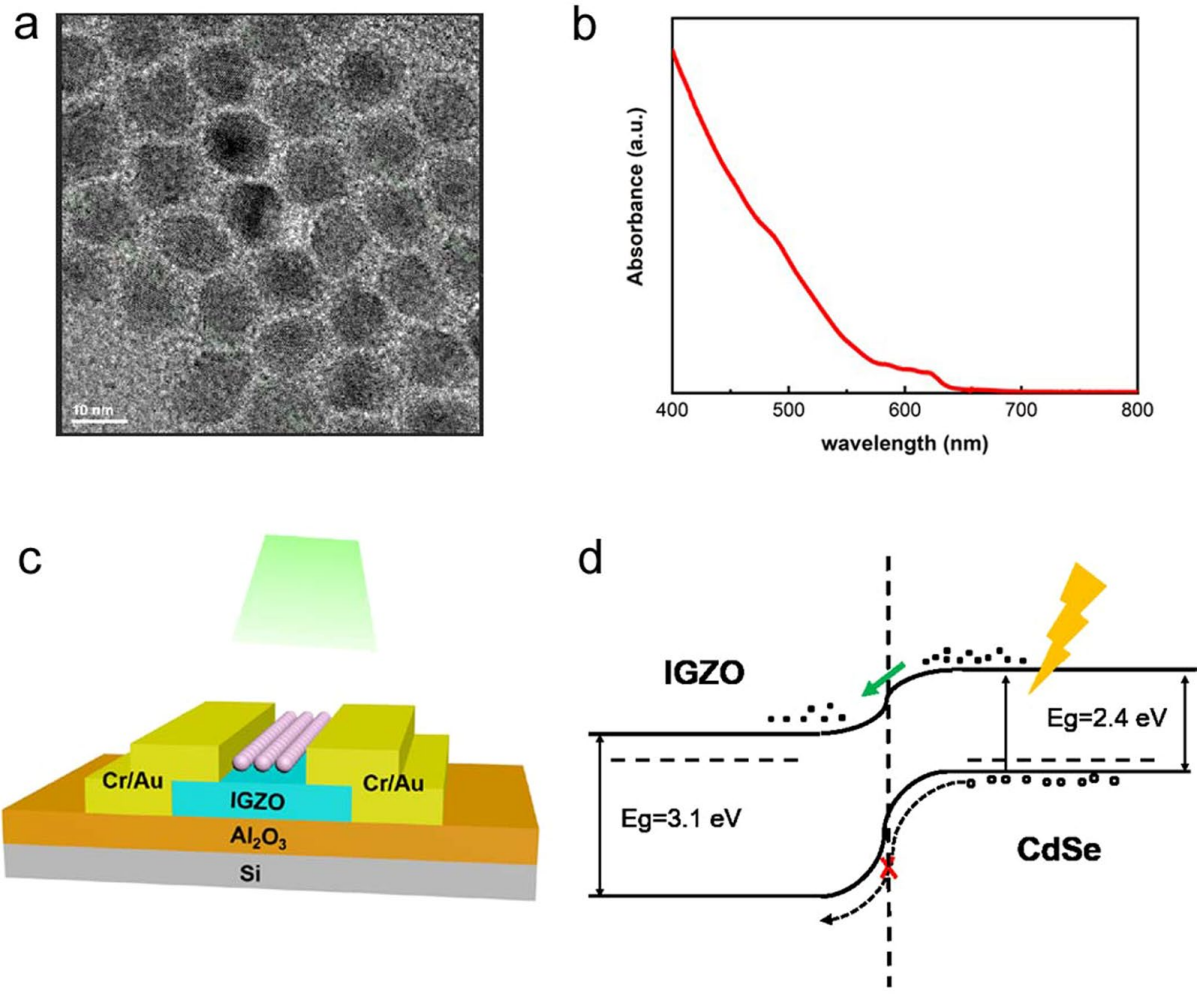
**a** HRTEM image of the CdSe QDs, **b** UV–vis absorption spectrum of CdSe QDs, **c** schematic diagram of the phototransistor with CdSe QDs/a-IGZO hybrid channel, **d** band diagram of the CdSe QD/a-IGZO hybrid channel

The CdSe QDs were used as light absorbers in the synaptic phototransistors. A high-resolution transmission electron microscopy (HRTEM) image of the as-synthesized CdSe QDs shows distinct crystalline lattice fringes with an average diameter of 15 nm, as shown in Fig. 1a. The ultraviolet–visible (UV–vis) absorption spectrum of the CdSe QDs has a cut-off wavelength of ~ 650 nm, which is much longer than that of a pure IGZO thin film for ~ 400 nm, as shown in Fig. 1b. A schematic diagram of the phototransistor is shown in Fig. 1c. Highly doped p-type Si serves as both the substrate and the gate electrode, Cr/Au electrodes act as the source and drain electrodes, and the CdSe QD/IGZO hybrid film is used as the channel layer. This hybrid film not only enhances the optical absorption of these devices, but also improves the charge separation efficiency of the photogenerated carriers in the CdSe QDs. The band diagram for the hybrid channel film is plotted in Fig. 1d. When illuminated using visible light, the CdSe QDs absorb the photons and produce photogenerated electron–hole pairs. The photogenerated electrons tend to inject into the IGZO film and change the film’s conductivity, while the photogenerated holes remain in the QDs because of the high valence band barrier. Therefore, enhanced photoelectric properties should be observed in the phototransistor.

**Fig. 2:**
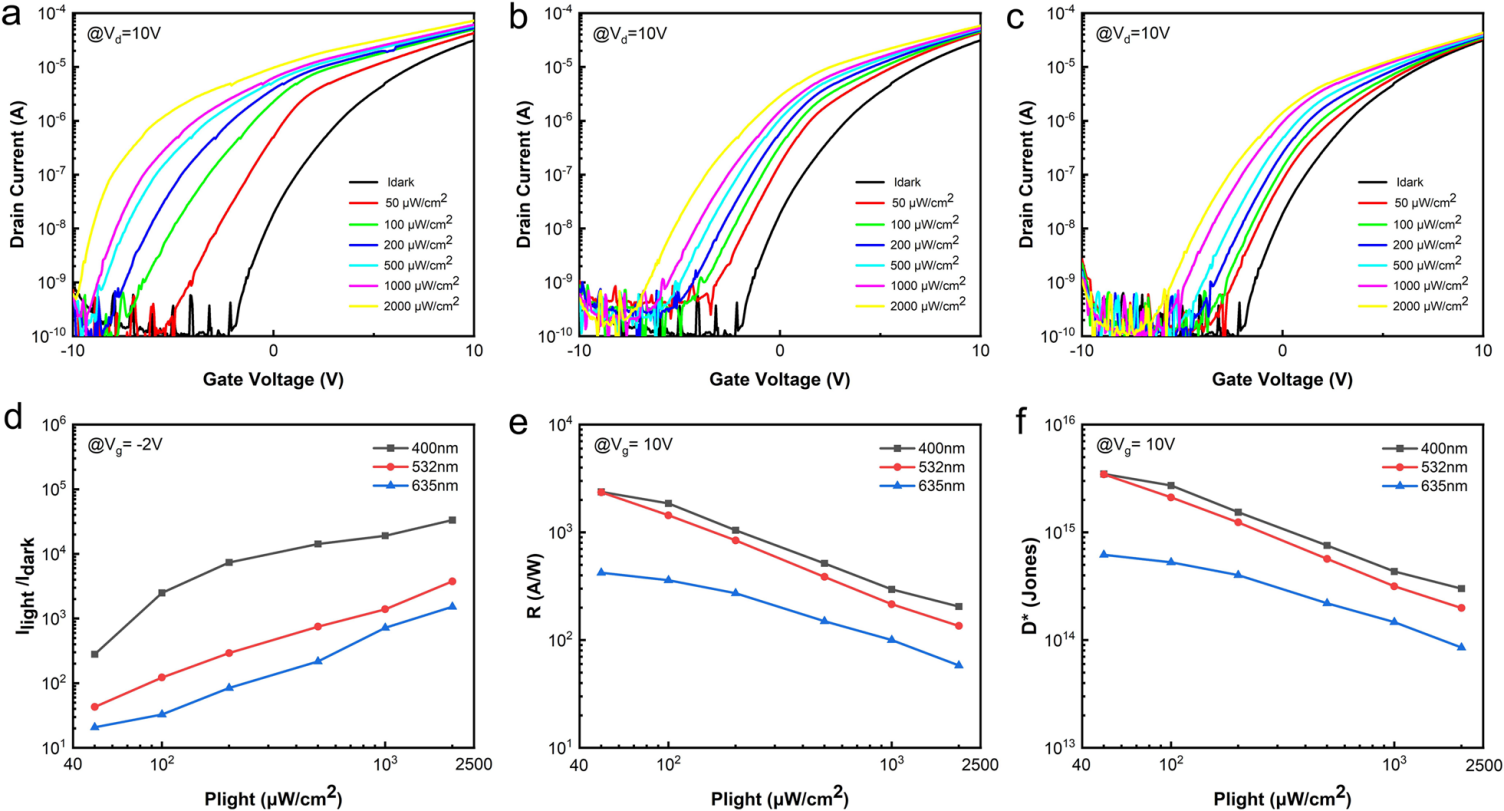
**a**–**c** Transfer curves of as-fabricated synaptic transistor measured in dark and under illumination by monochromatic light at different wavelengths ranging from 400 to 635 nm. **d** The *I*_light_/*I*_dark_ ratio measured at *V*_d_ = 10 V and *V*_g_ =  − 2 V, **e** responsivity and f) detectivity of the as-fabricated synaptic transistor as functions of the incident light power intensity, measured at *V*_d_ = *V*_g_ = 10 V

The photoelectric performance characteristics of the CdSe QD/IGZO hybrid channel phototransistors were investigated under illumination by monochromatic light at different wavelengths ranging from 400 to 635 nm. Figure 2a shows the transfer curves of the phototransistor as measured in the dark and under illumination by 400 nm monochromatic light. The device shows a strong photoelectric response when illuminated by the 400 nm light, even when the light intensity is as low as 50 μW/cm^2^. The drain current increases with increasing incident light power because higher light power stimulates the generation and injection of more photoelectrons into the IGZO film, and leads to an increase in the film’s conductance. Similar phenomena were observed in devices illuminated by incident light at 532 nm and 635 nm, as shown in Fig. 2b, c, respectively. Furthermore, the photoelectric properties of the devices were seen to decrease significantly as the wavelength of the incident light increased. Figure 2d shows the *I*_light_/*I*_dark_ ratios of these devices as functions of the incident light intensity. For this measurement, the drain voltage was set at *V*_d_ = 10 V and the gate voltage was set to *V*_g_ =  − 2 V. The result shows that the *I*_light_/*I*_dark_ ratio increases with increasing light power intensity or increasing photon energy. These results are in close agreement with the absorption spectrum of CdSe shown in Fig. 1b, indicating that the visible light responses of the hybrid phototransistors originate from the CdSe QDs.

The photoresponsivity (*R*) and detectivity (D^*^) are two key parameters for the photoelectric devices. The *R* indicants the photoelectric conversion capability of the phototransistors, which can be expressed as [17]:

$$R = \frac{{I_{{{\text{photo}}}} }}{P \cdot S}$$

where *P* means the incident light power intensity, and *S* is the channel area of the devices. The D^*^ is determined by the noise current, which is consisted of shot noise from dark current, Johnson noise and flicker noise. Since the D^*^ is often stated in the shot-noise limit in which only shot noise from dark current is considered, the D^*^ can be expressed as [18]:

$$D^{*} = R\sqrt {\frac{S}{{2eI_{{{\text{dark}}}} }}}$$

Figure 2e, f shows the *R* and D^*^ values of these devices as functions of the incident light power intensity, where the darin and gate voltages were set as *V*_d_ = *V*_g_ = 10 V. A high *R* values over 2.4 × 10^3^ A/W and a high D^*^ over 3.5 × 10^15^ are observed when the incident light wavelength is shorter than 532 nm. These results indicate that the hybrid channel phototransistors are highly sensitive to blue and green lights.

**Fig. 3:**
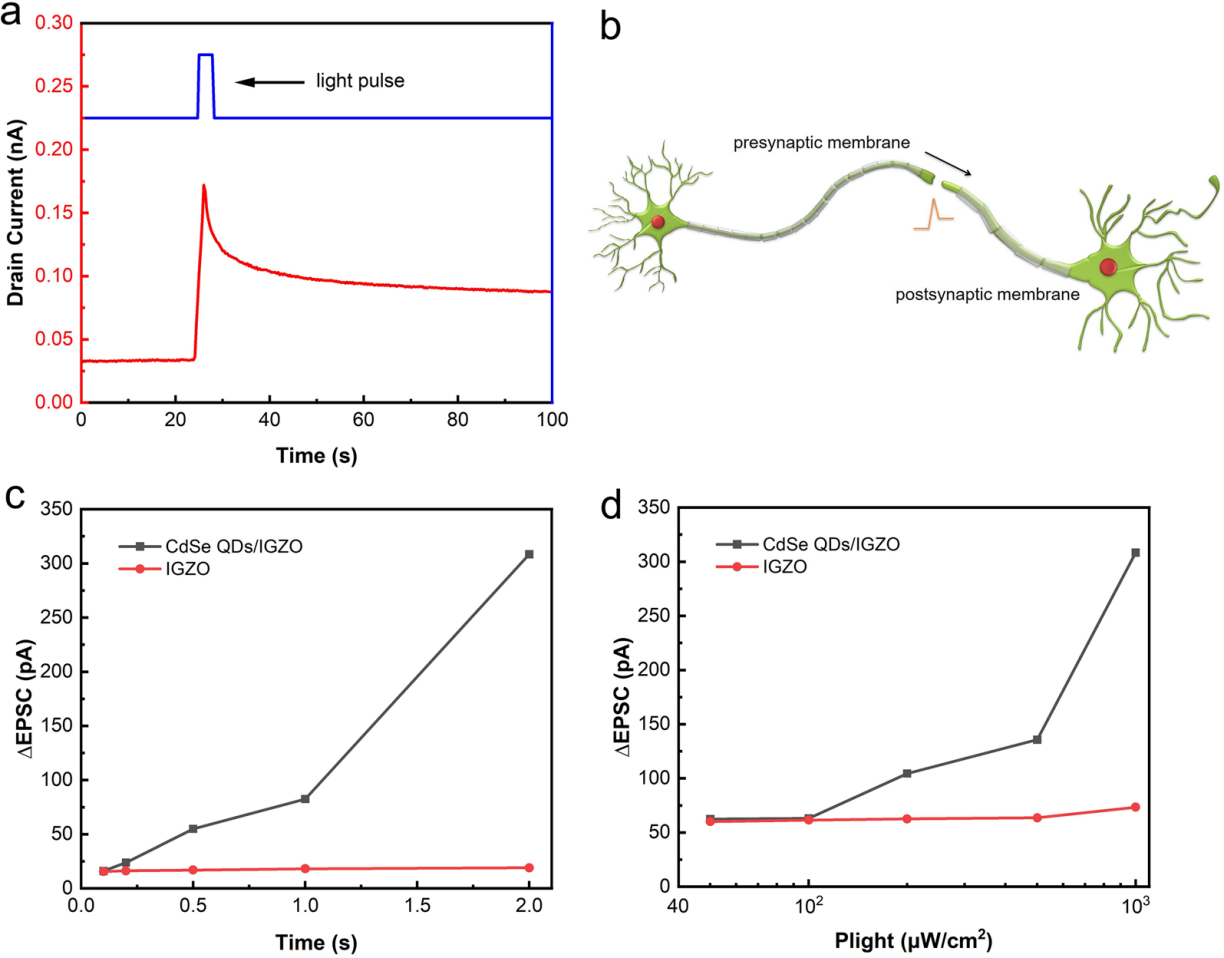
**a** EPSC performance of the synaptic transistor stimulated by visible light pulse (532 nm, 1 mW/cm^2^, 2 s). **b** Schematic diagram of the signal transmission process between biological synapses. **c** and **d** The of EPSC gain as functions of incident light pulse width and light power intensity in hybrid channel transistor and IGZO transistor

These CdSe QD/IGZO hybrid channel phototransistors not only present excellent photoelectric properties, but also show typical photoelectric synaptic behaviors because of their unique channel design. In biological neural networks (BNNs), the presynapse can release excitatory neurotransmitters under stimulation, and these neurotransmitters can bind the receptors on the postsynapse, resulting in an excitatory postsynaptic current (EPSC). The EPSCs are summed in the postneuron, which determines the fire of the neuron [19]. Figure 3a shows the EPSC behavior of the phototransistors when triggered by a visible light pulse (532 nm, 1 mW/cm^2^, 2 s) and measured at a constant drain voltage of 1 V and a gate voltage of 0.1 V. The current increases abruptly under illumination by the visible light pulse, and then decreases slowly, corresponding to a relaxation equation after the light pulse is switched off. This phenomenon originates from the spatial separation of the photogenerated carriers in the hybrid channel. When illuminated by the incident light, the photoelectrons that are generated in the CdSe QDs are injected into the a-IGZO film, while the photogenerated holes are trapped in the QDs because of the high valence band barrier. These electrons enhance the conductivity of the IGZO film significantly and cause a sudden increase in the drain current. After the light source is turned off, the separated electrons and holes recombine slowly, resulting in a slow decay in the drain current. This behavior is very similar to that of biological synapses, as illustrated in Fig. 3b. Figure 3c shows the increase in the EPSC (ΔEPSC) as a function of the presynaptic light pulse width. A series of 532 nm monochromatic light pulses with different pulse widths ranging from 50 ms to 2 s were used to investigate the influence of the light pulse width on ΔEPSC. The ΔEPSC value was observed to increase gradually with increasing presynaptic light pulse width. Similarly, an increase in the ΔEPSC value was also observed with increasing incident light pulse intensity when the intensity varied from 50 μW/cm^2^ to 1 mW/cm^2^, as shown in Fig. 3d. These results are related to the enhanced carrier generation in CdSe QDs when illuminated using light pulses with greater widths or higher intensities. For comparison, the same investigation conditions were also applied to an IGZO thin-film transistor (TFT), where the EPSC values showed negligible changes. This indicates that the photoelectric behavior of these hybrid channel transistors under visible light illumination originates from the CdSe QDs.

**Fig. 4:**
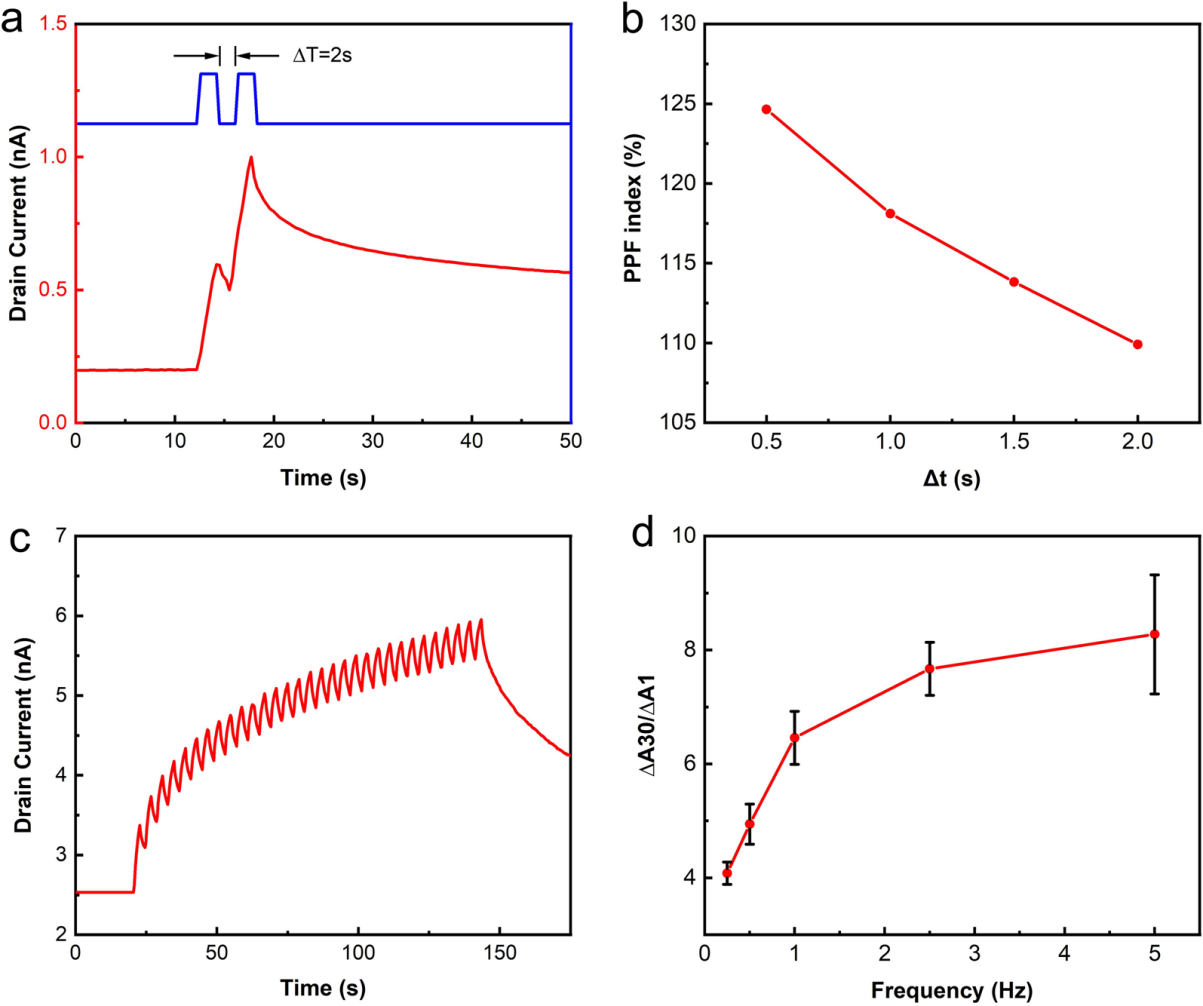
**a** A typical PPF behavior of the CdSe QDs/IGZO hybrid channel synaptic phototransistor. **b** The PPF index as a function of the light pulse interval (532 nm, 1 mW/cm^2^). **c** the LTP behavior of the phototransistor measured by applying 30 continuous light pulses (532 nm, 1 mW/cm^2^, 2 s). **d** The EPSC gain ratio (∆*A*30/∆*A*1) as a function of the light pulse frequency

PPF is a phenomenon that occurs in neuroscience, where the peak value of the EPSC caused by the second spike is higher than that caused by the first spike. The PPF is an important parameter that is used to assess the short-term plasticity (STP) of synaptic devices. Figure 4a shows the typical PPF behavior of the CdSe QD/IGZO hybrid channel synaptic phototransistor. Two EPSC peaks are observed when two successive visible-light pulses (532 nm, 1 mW/cm^2^, 2 s) are applied to the device channel. The second EPSC triggered by the second light pulse is significantly higher than that triggered by the first pulse. This phenomenon is very similar to biological PPF behavior. The PPF index is defined using the following formula [18]:

$${\text{PPF}} = \frac{A1}{{A2}} \times 100\%$$

where *A*1 and *A*2 are the peak values of the first and second EPSCs, respectively. Figure 4b presents the PPF index of the CdSe QD/IGZO hybrid channel synaptic phototransistor as a function of the time interval between successive pulses. The PPF index decreases as the time interval increases.

In addition, the LTP of the phototransistor was also evaluated by application of 30 continuous light pulses (532 nm, 1 mW/cm^2^, 2 s) to the device. An obvious increase in the EPSC can be observed after the 30 continuous light pulses, as illustrated in Fig. 4c. The increase in the value of the EPSC (Δ*A*30) is almost four times higher than that stimulated by the first pulse (Δ*A*1). The EPSC gain (defined as Δ*A*30/Δ*A*1) is plotted as a function of the light pulse frequency, as shown in Fig. 4d. The EPSC gain increases as the light pulse frequency increases from 0.25 to 2 Hz. This behavior is consistent with that of biological synapses.

## Conclusion

In this work, novel visible-light-stimulated synaptic phototransistors based on CdSe QDs and IGZO were demonstrated successfully. The type-II heterojunction consisting of CdSe QDs and an IGZO film can enhance the charge separation efficiency of the photogenerated carriers significantly, and also improves the photoresponsivity of the phototransistor. Furthermore, typical photoelectric synaptic behaviors have also been realized successfully in this device, including EPSC, PPF, STP and LTP. This work provides a simple but effective way to fabricate visible-light-stimulated artificial synapses, which may represent a new direction for the development of neuromorphic devices.

## Data Availability

All datasets are presented in the main paper and freely available to any scientist wishing to use them for non-commercial purposes, without breaching participant confidentiality.

## Abbreviations

CdSe QD: CdSe quantum dot
a-IGZO: Amorphous In–Ga–Zn–O
*I*_photo_/*I*_dark_: Photocurrent-to-dark current
R: Photoresponsivity
*D**: Detectivity
EPSC: Excitatory postsynaptic current
PPF: Paired-pulse facilitation
LTP: Long-term plasticity
SC-1: Standard clean
TMA: Trimethylaluminum
ALD: Atomic layer deposition
DPPSe: Diphenylphosphine selenide
ODE: Octadecene
HRTEM: High-resolution transmission electron microscopy
UV–vis: Ultraviolet–visible
TFT: Thin film transistor
STP: Short-term plasticity
